# The *KRAS-*Variant and miRNA Expression in RTOG Endometrial Cancer Clinical Trials 9708 and 9905

**DOI:** 10.1371/journal.pone.0094167

**Published:** 2014-04-14

**Authors:** Larissa J. Lee, Elena Ratner, Mohamed Uduman, Kathryn Winter, Marta Boeke, Kathryn M. Greven, Stephanie King, Thomas W. Burke, Kelly Underhill, Harold Kim, Raleigh J. Boulware, Herbert Yu, Vinita Parkash, Lingeng Lu, David Gaffney, Adam P. Dicker, Joanne Weidhaas

**Affiliations:** 1 Department of Radiation Oncology, Brigham and Women’s Hospital/Dana-Farber Cancer Institute, Boston, Massachusetts, United States of America; 2 Department of Gynecologic Oncology, Yale University, New Haven, Connecticut, United States of America; 3 Interdepartmental Program in Computational Biology and Bioinformatics, Yale University, New Haven, Connecticut, United States of America; 4 Statistical Center, Radiation Therapy Oncology Group (RTOG), Philadelphia, Pennsylvania, United States of America; 5 Department of Radiation Oncology, Wake Forest University Baptist Medical Center, Winston-Salem, North Carolina, United States of America; 6 Department of Surgical Oncology, Fox Chase Cancer Center, Philadelphia, Pennsylvania, United States of America; 7 Department of Gynecologic Oncology and Reproductive Medicine, University of Texas MD Anderson Cancer Center, Houston, Texas, United States of America; 8 Department of Radiation Oncology, Benefis Sletten Cancer Institute, Great Falls, Montana, United States of America; 9 Department of Radiation Oncology, Wayne State University Karamanos Cancer Center, Detroit, Michigan, United States of America; 10 Radiation Oncology, South Carolina Oncology Associates, Columbia, South Carolina, United States of America; 11 Department of Radiation Oncology, Thomas Jefferson University Hospital, Philadelphia, Pennsylvania, United States of America; 12 Department of Chronic Disease and Epidemiology, Yale University, New Haven, Connecticut, United States of America; 13 Cancer Epidemiology Program, University of Hawaii Cancer Center, Honolulu, Hawaii, United States of America; 14 Department of Therapeutic Radiology, Yale University, New Haven, Connecticut, United States of America; 15 Department of Radiation Oncology, University of Utah Huntsman Cancer Hospital, Salt Lake City, Utah, United States of America; 16 Department of Pathology, Yale University, New Haven, Connecticut, United States of America; Baylor College of Medicine, United States of America

## Abstract

**Objective:**

To explore the association of a functional germline variant in the 3′-UTR of *KRAS* with endometrial cancer risk, as well as the association of microRNA (miRNA) signatures and the *KRAS*-variant with clinical characteristics and survival outcomes in two prospective RTOG endometrial cancer trials.

**Methods/Materials:**

The association of the *KRAS*-variant with endometrial cancer risk was evaluated by case-control analysis of 467 women with type 1 or 2 endometrial cancer and 582 age-matched controls. miRNA and DNA were isolated for expression profiling and genotyping from tumor specimens of 46 women with type 1 endometrial cancer enrolled in RTOG trials 9708 and 9905. miRNA expression levels and *KRAS-*variant genotype were correlated with patient and tumor characteristics, and survival outcomes were evaluated by variant allele type.

**Results:**

The *KRAS*-variant was not significantly associated with overall endometrial cancer risk (14% controls and 17% type 1 cancers), although was enriched in type 2 endometrial cancers (24%, p = 0.2). In the combined analysis of RTOG 9708/9905, miRNA expression differed by age, presence of lymphovascular invasion and *KRAS*-variant status. Overall survival rates at 3 years for patients with the variant and wild-type alleles were 100% and 77% (HR 0.3, p = 0.24), respectively, favoring the variant.

**Conclusions:**

The *KRAS-*variant may be a genetic marker of risk for type 2 endometrial cancers. In addition, tumor miRNA expression appears to be associated with patient age, lymphovascular invasion and the *KRAS-*variant, supporting the hypothesis that altered tumor biology can be measured by miRNA expression, and that the *KRAS-*variant likely impacts endometrial tumor biology.

## Introduction

The clinical and biologic heterogeneity of endometrial cancer has been well recognized since the description of two major subtypes by Bokhman in 1983[1]. Type 1 endometrial cancer, the endometrioid subtype, is associated with unopposed estrogen exposure, chronic anovulation, nulliparity and obesity. Type 2 endometrial cancer includes the less common non-endometrioid subtypes of uterine papillary serous and clear cell carcinoma. Type 2 cancers are frequently seen in older women, arise in atrophic endometrium, and are not estrogen responsive. The two-tiered classification of endometrial cancer based on clinical and pathologic factors is also supported at a molecular level. Further molecular classification has recently been put forth by the publication of The Cancer Genome Atlas[2]. Loss of the tumor suppressor *PTEN* has been reported in 30–50% of type 1 cancers[3]–[5], but is rarely observed in the serous or clear cell subtypes[6]. The proto-oncogene HER2/neu, a trans-membrane growth factor receptor, is commonly amplified or over-expressed in type 2 cancers[7], [8]. Gene expression profiling by microarray analysis has also confirmed the presence of distinct endometrial cancer subtypes[9]. Other characteristic molecular alterations include microsatellite instability[10] and mutation of *beta-*catenin[11] in endometrioid endometrial carcinomas, and loss of E-cadherin expression and p53 mutations in serous cancers[5]. Tumor acquired mutations in *KRAS* have been identified in 15–30% of type 1 endometrial cancers but are rarely observed (0–5%) in type 2 cancers[12]. The prevalence of tumor-acquired *KRAS* mutations has been variably associated with stage, grade and survival in endometrial cancer[13]–[16]. *KRAS* mutations have also been detected in endometrial hyperplasia, and may represent an early event in tumorigenesis for type 1 endometrial cancers.

While adjuvant therapy for type 2 endometrial cancers often involves chemotherapy, the role of combined chemotherapy and radiation therapy (RT) for high-risk and advanced stage type 1 cancers is more controversial. Women with type 1 uterine cancers with pathologic risk factors such as deep myometrial invasion, high tumor grade, cervical invasion and/or pelvic-confined extrauterine spread have a 15–30% risk of recurrence despite adjuvant pelvic radiotherapy (RT) [17]. The Radiation Therapy Oncology Group (RTOG) conducted two prospective, multi-institutional trials (9708 and 9905) that were designed to evaluate the feasibility and efficacy of concurrent chemotherapy and post-operative RT for women with International Federation of Gynecology and Obstetrics (FIGO) Stages IC-IIIC, high-risk, type 1 endometrial cancer. Given the reported response rates of 30–35% with chemotherapy alone, these trials were conducted to evaluate the safety and survival outcomes for a combined modality approach for high-risk and advanced stage endometrial cancer. RTOG 9905, the randomized comparison of adjuvant chemoradiotherapy versus RT alone, was closed early due to poor accrual and therefore survival endpoints were not analyzed. Nevertheless, tissue was collected for the exploration of novel biomarkers and clinical correlations.

MicroRNAs (miRNAs) are a class of small non-coding RNA that inhibit gene expression of downstream targets by binding complementary sites in 3′ untranslated regions (UTR) of messenger RNA. As global gene regulators, miRNAs function as a novel class of oncogenes or tumor suppressors depending on the cellular context. Alterations in miRNA expression levels have been implicated in oncogenesis as well as tumor biology in virtually all cancers[18]. Distinct miRNA expression signatures have also been described for the type 1 and type 2 endometrial cancers[19], supporting their ability to reflect inherent tumor biology. In addition, inherited variants in miRNA binding sites in oncogenes have been shown to predict cancer risk, tumor biology and altered miRNA signatures[20].

The first example of a functional miRNA binding site mutation is a variant allele in the 3′-UTR of *KRAS* (rs61764370), a germline mutation found to disrupt *let-7* binding, increase *KRAS* expression[21] and predict cancer risk[21]–[24]. This mutation has additionally been associated with altered gene and miRNA expression in tumors[21]–[23], [25]. Interestingly, the *KRAS-*variant predicts ovarian cancer risk in post-menopausal women[22], triple negative breast cancer in pre-menopausal women[22], and aggressive breast tumor biology in post-menopausal women with a history of hormone replacement therapy[26], suggesting that there is a likely impact of estrogen on the tumor-associated function of the *KRAS-*variant. In addition, the *KRAS-*variant appears to predict cancer biology in all cancers thus far studied, including those for which it does not appear to predict increased risk[27]–[28].

The objectives of this study were multifold: 1) to determine if the *KRAS-*variant is associated with endometrial cancer risk; 2) to evaluate the association of miRNA expression signatures with clinical features in tumor specimens from RTOG 9708 and 9905; and 3) to determine whether the *KRAS*-variant is associated with endometrial cancer biology by evaluating both clinical and miRNA expression associations in these same trials.

## Materials and Methods

### Case-control Data

The subjects of the case-control study were Connecticut residents diagnosed with primary endometrial cancer between October 2004 and September 2008. Study staff of the Rapid Case Ascertainment arm of the Yale Cancer Center visited the Connecticut general hospitals to determine case eligibility and identifying information. Controls were identified by random digit dialing. Blood samples or saliva specimens were collected from these study subjects. From collected specimens, DNA was isolated using MagNA pure nucleic acid kit (Roche diagnostics, Indianapolis, IN) for the buffy-coat blood cells, and Oragene kit (DNA Genotek Inc, Canada) for saliva samples. The presence of the *KRAS*-variant was detected using a TaqMan PCR assay to identify the wild-type (T) or variant (G) allele using allele-specific probes, as previously described [21]. Due to the minor allele frequency, the heterozygous (TG) and homozygous (GG) forms were combined for analysis to compare to the wild-type allele (TT).

### Ethics Statement for Case-control Data

This protocol was approved by the Yale Human Investigations Committee and State of Connecticut Department of Public Health Human Investigation Committee. Certain data used in this study were obtained from the Connecticut Tumor Registry in the Connecticut Department of Public Health.

### RTOG 9708 and 9905

RTOG 9708 was a single-arm phase II study of adjuvant RT combined with cisplatin and paclitaxel chemotherapy, which enrolled 45 patients with type I FIGO 1988 Stage IC-IIIC endometrioid adenocarcinoma of the uterus. The successor trial, RTOG 9905, was a randomized two-arm phase III study of adjuvant RT with or without the same chemotherapy regimen, which enrolled 42 patients. All patients enrolled in the two studies started adjuvant radiation therapy within 8 weeks of surgery (total abdominal hysterectomy and bilateral salpingo-oophorectomy). Eligible patients had grade 2 or 3 adenocarcinoma with greater than 50% myometrial invasion, cervical stromal involvement or pelvic-confined extrauterine disease and/or positive peritoneal cytology. Patients with non-endometriod histology, such as papillary serous or clear cell, were excluded from these studies.

Adjuvant treatment included pelvic RT to a dose of 45–50.4 Gy followed by vaginal brachytherapy. Cisplatin (50 mg/m2) was delivered concurrently on days 1 and 28. Following the completion of RT, patients received 4 cycles of adjuvant cisplatin (50 mg/m^2^) and paclitaxel (160–175 mg/m^2^) given every 4 weeks. Patients were seen in follow-up every 4 months for 2 years, then every 6 months for 1 year and annually thereafter. A Pap smear and chest X-ray were performed every year.

### Ethics Statement for RTOG 9708 and 9908

Written informed consent was obtained from all patients prior to enrollment. The clinical outcome and toxicity data for RTOG 9708 have been previously published [17], [29]. RTOG 9905 was terminated early due to poor accrual and follow up was discontinued after at least 12 months of follow up for each patient.

### Isolation of DNA and RNA and Testing of the *KRAS*-variant and miRNAs

Tumor specimens from paraffin-embedded formalin-fixed blocks were microdissected after identification of areas with sufficient tumor cellularity by a pathologist. Tumor sections were de-paraffinized using Xylene and DNA was isolated for genotyping of the *KRAS*-variant as described above. Total RNA was isolated from paraffin-embedded tumor specimens using the Ambion Recover All kit (Life Technologies, Grand Island, NY) per manufacturer’s instructions. miRNA was run on the ABI Taqman TLDA platform. Samples were genotyped for the *KRAS-*variant. The data was in Hardy-Weinberg equilibrium.

### Statistical Analysis

Statistical analysis was performed using the statistical software JMP, v. 8.0.1 (SAS Institute, Cary, NC). For the case-control analysis, the prevalence of the variant allele (TG/GG) was compared for cases with endometrial cancer and population controls using the Fisher exact test. Clinical, pathologic and treatment characteristics of patients in the RTOG trials were compared by allele type (wild-type vs. variant) using a t-test or the Fisher exact test. Actuarial estimates of disease-free survival (DFS) and overall survival (OS) were calculated using the Kaplan-Meier method. The cumulative incidence method was used to estimate the rates of local-regional failure (LRF) and distant failure (DF). OS was calculated from the date of randomization until death from any cause. DFS was defined as the interval from the date of randomization until the development of distant metastases, local-regional failure or death from any cause. LRF was defined as primary recurrence or progression, vaginal recurrence and/or nodal recurrence or progression. DF was defined as distant metastases and/or para-aortic failure. Univariate analysis was performed using Cox proportional hazards models. Estimated prevalence rates for the variant allele were used for power calculations. A type 1 error (α) of less than 0.05 was considered statistically significant.

For miRNA expression data, all pre-processing and statistical analysis was performed in the statistical programming environment R, using customized functions and the Bioconductor limma package. Each sample was normalized separately using the eight endogenous control RNAs, and then the intensities were scaled across the samples to have similar distributions. Statistically significant changes in miRNA expression between samples grouped by age, race, presence of lymphovascular space invasion, DFS and *KRAS*-variant status were determined using limma with p<0.05 adjusted by a Bonferroni correction, and an absolute fold-change ≥2. Hierarchical clustering was based on Euclidean distance metric and Ward’s linkage.

## Results

### The *KRAS*-variant and Endometrial Cancer Risk

In the case-control study, to evaluate the role of the *KRAS-*variant in predicting uterine cancer risk, allele data was obtained for 583 population controls, 430 patients with type 1 endometrial cancer and 37 patients with type 2 cancer. The prevalence of the variant allele was 13.9% among controls and 16.5% for patients with endometrial cancer (p = 0.29). As shown in **Table 1**, the prevalence of the variant appeared higher in patients with type 2 uterine cancer of non-endometrioid histology (24.3%) compared to type 1 endometrial cancer (16.7%) or population controls (13.9%). However, none of the differences reached statistical significance likely due to the small number of type 2 cancers in this study.

**Table 1:** Prevalence of *KRAS*-variant in a control population and in patients with types 1 and 2 endometrial cancer.

KRAS-variant	Control	Type 1 cancer	Type 2 cancer
Non-variant homozygotes	501 (86.1%)	360 (83.3%)	28 (75.7%)
Mutant/heterozygotes	81 (13.9%)	70 (16.7%)	9 (24.3%)
p-value	0.19		

### MiRNA Expression in RTOG 9708 and 9905

**Figure 1:**
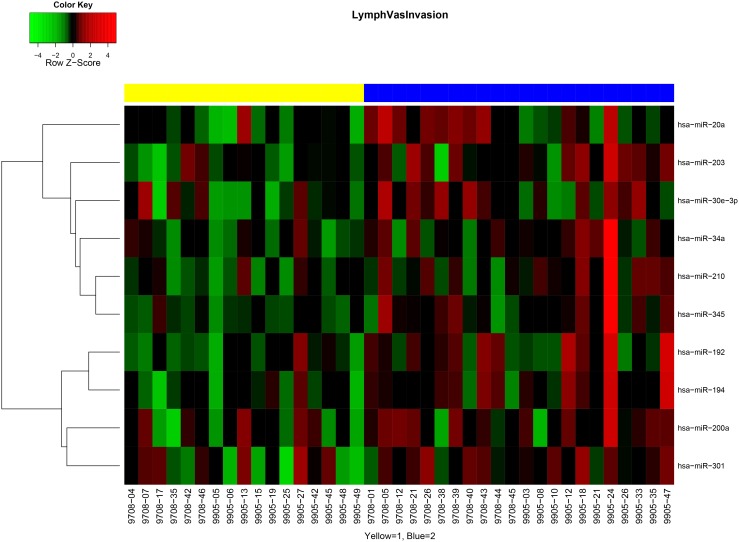
MiRNA signatures differ in tumors with lymphovascular invasion (LVI). Yellow represents patients with LVI, and blue represents patients without LVI.

**Figure 2:**
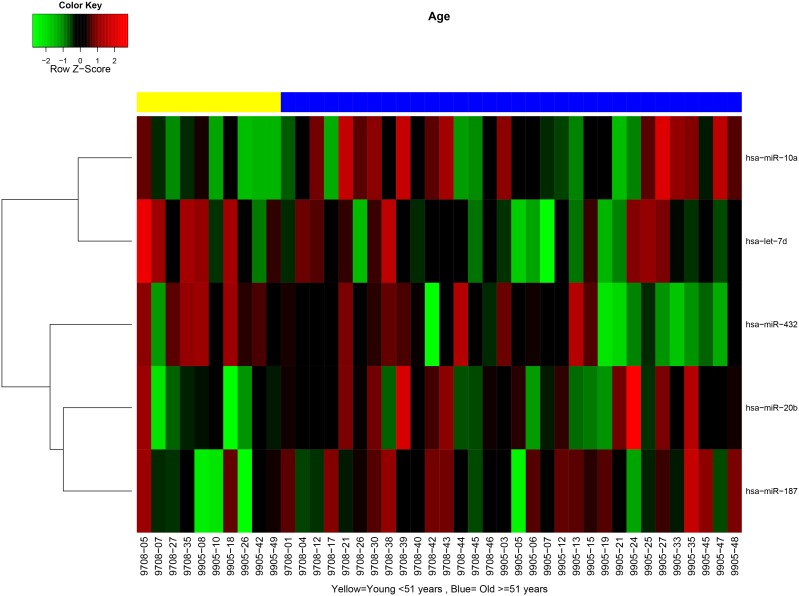
MiRNA signatures differ in tumors from women over and under 51 years of age. Yellow represents patients younger than 51 years of age, and blue represents patients equal to or older than 51 years of age.

MiRNA expression patterns were evaluated from tumor specimens of patients enrolled in RTOG 9708 and 9905 and tested for association with clinical and pathologic characteristics. We found no significant differences in miRNA expression between samples from the two trials (data not shown), therefore the samples were combined for the subsequent analysis. miRNA expression patterns differed between tumors with lymphovascular invasion (LVI) versus those without. Differentially expressed miRNAs (p value<0.05 and fold-change>2) included miR-194, miR-192, miR-203, miR-345, miR-30e-3p, miR-210 and miR-301 (**Table S1**, **Figure 1**), all of which were overexpressed in tumors with LVI present. Similarly, there were differences in miRNA expression based on patient age: tumors in older women (≥51 years) showed overexpression of miR-20b, miR-10a, and miR-187 and underexpression of miR-432 and let-7d (**Table S2**, **Figure 2**) compared with tumors of younger women (<51 years).

### Clinical Associations of the *KRAS*-variant in Trials RTOG 9708 and 9905

Allele data were available for 46 of 87 evaluable patients (53%) enrolled in RTOG 9708 and 9905. The availability of allele data was similar for the two trials, which included 22 patients (49%) from 9708 and 24 patients (57%) from 9905. The prevalence of the *KRAS*-variant was 22% overall, and was 18% (n = 4) and 25% (n = 6) from 9708 and 9905, respectively. The *KRAS-*variant was found in only 1 of 11 (10%) women younger than 51 years of age at the time of uterine cancer diagnosis, and in 9 of 35 (28%) women who were older, although these prevalence rates were not statistically different (p = 0.41), likely due to small sample size.

The median follow-up time for patients with allele data in the combined analysis was 29.3 months (min–max, 6.8–124.1 months). Patient characteristics, including age, ECOG performance status, and race, were not significantly different between women with and without the *KRAS-*variant (**Table 2**). The 1988 FIGO stage distribution, histology, depth of myometrial invasion greater than 50% and the presence of LVI were also similar between the groups.

**Table 2:** Clinical and pathologic characteristics of 46 patients with allele data from RTOG trials 9708 and 9905.

		Wildtype TT	Variant TG or GG	p-value
		(n = 36)	(n = 10)	
Age	Median (min–max)	61 years (36–81)	56 years (39–68)	0.16
Race	White	29 (81%)	9 (90%)	0.66
	Non-white	7 (19%)	1 (10%)	
ECOG score	0	28 (78%)	8 (80%)	1.0
	1–2	8 (22%)	2 (20%)	
Histology	Adenocarcinoma	31 (86%)	9 (90%)	0.74
	Adenosquamous	3 (8%)	1 (10%)	
	Other	2 (6%)	0	
Stage	IB/IC/IIA/IIB	26 (72%)	6 (60%)	0.46
	IIIA/IIIC	10 (28%)	4 (40%)	
FIGO grade	1–2	19 (53%)	6 (60%)	0.73
	3	17 (47%)	4 (40%)	
MMI	>50%	31 (86%)	8 (80%)	0.64
LVI		19 (53%)	5 (50%)	0.88

Key: min = minimum; max = maximum; ECOG score = Eastern Cooperative Oncology Group performance status score; FIGO = International Federation of Gynecology and Obstetrics; MMI = myometrial invasion; LVI = lymphovascular invasion.

The 3-year OS rates were 100% for the *KRAS-*variant patients and 77% (95% confidence interval [CI]: 58%–87%) for the non-variant patients (hazard ratio [HR]  = 0.3, 95% CI: 0.04–2.29, p = 0.24). One of 10 patients (10%) with the *KRAS-*variant had LRF and 2 (20%) had DF compared to 7 (19%) and 13 (36%), respectively, among patients with the non-variant allele. The HRs for LRF and DF were 0.45 (95% CI: 0.05–3.65, p = 0.45) and 0.44 (95% CI: 0.10–1.97, p = 0.28) for variant carriers. These trends were not significant, perhaps because the study power was only 14–41% to detect a significant difference in the clinical endpoints due to small sample size and limited follow-up.

### MiRNA Expression and the *KRAS*-variant in RTOG 9708 and 9905

**Figure 3:**
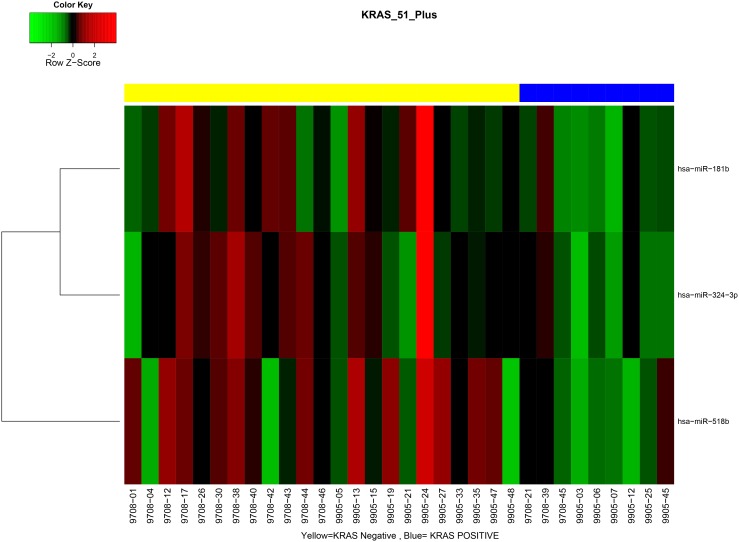
MiRNA signatures differ between tumors with and without the *KRAS-*variant. Yellow represents patients without the *KRAS-*variant, and blue patients with the *KRAS-*variant.

Because the *KRAS-*variant has been previously shown to be associated with altered gene as well as miRNA expression in tumors, we evaluated the miRNA signatures from tumors with and without the *KRAS-*variant. Furthermore, given that miRNA signatures differed between tumors based on patient age (cutpoint 51 years), and that most patients with the *KRAS-*variant were post-menopausal, we evaluated these differences only in tumors from patients older than 51 years. We found significant differences in miRNA signatures between tumors from patients with the *KRAS-*variant and those without the *KRAS-*variant. These included lower expression of miR-181b, miR-324–3p and miR-518b in *KRAS-*variant patients’ tumors (**Table 3**, **Figure 3**).

**Table 3:** miRNA expression in *KRAS-*variant negative versus positive tumors.

ID	logFC	p-Value	Adjusted p-Value	m.e. KRAS Negative	m.e. KRAS Positive
hsa-miR-181b	–1.2486	0.0326	0.6593	1.9609	3.2095
hsa-miR-324-3p	–1.1496	0.0432	0.6593	4.2346	5.3842
hsa-miR-518b	–1.0684	0.0452	0.6593	6.5858	7.6542

Key: logFC = log fold change; m.e. = mean expression.

## Discussion

This study represents the first analysis of the association of the *KRAS-*variant with endometrial cancer. Although the *KRAS*-variant was not a predictor of overall endometrial cancer risk, it was enriched in patients with type 2 cancers, with a prevalence of 24.3%. In addition, in two prospective RTOG endometrial cancer trials, miRNA signatures as well as the *KRAS-*variant were associated with distinct clinical features. miRNA signatures were different between tumors with or without LVI, between tumors in pre- versus post-menopausal patients, and between *KRAS-*variant patients and non-variant patients. Although RTOG 9708 and 9905 were not powered to detect statistically significant associations between the *KRAS*-variant and outcome, the trend was that the *KRAS-*variant was associated with better outcome, although confirmation of this hypothesis requires testing in a larger study with more statistical power.

Although the association of miRNA expression and altered clinical features of a tumor has been well documented, it may seem surprising that the *KRAS*-variant, a germ-line, non-protein coding sequence mutation that disrupts miRNA binding, predicts altered miRNA signatures in a tumor and also alters prognosis. However, the association of the *KRAS-*variant with altered miRNA signatures has been found in every tumor in which it has been analyzed, including lung cancer[21], melanoma[10], head and neck cancer[30] and triple negative breast cancer[23].

The association of the *KRAS-*variant with prognosis across tumor types has also been widely reported. In a cohort of 344 patients with head and neck cancer, although an association with cancer risk was not observed, variant carriers had a worse clinical outcome when adjusted for age and stage (HR 1.6, 95% CI 1.0–2.5) [30], with lower survival rates most pronounced among patients with oral cavity cancer compared to pharyngeal and laryngeal sites. Several studies have found that the *KRAS*-variant predicts altered response to cetuximab in patients with metastatic colorectal cancer[28], [31]. In ovarian cancer, the *KRAS-*variant predicts poor outcome due to platinum resistance[23]. Platinum resistance was also found in patients receiving chemotherapy alone for metastatic head and neck cancer (Chung, submitted). Platinum resistance is particularly interesting in the context of the RTOG trials examined in this study, where a proportion of patients received cisplatin delivered concurrently with radiation and in conjunction with paclitaxel. One might hypothesize that the addition of radiation to platinum agents might overcome the associated resistance, a hypothesis that is currently being evaluated in additional RTOG trials.

Among *KRAS*-variant positive and negative tumors in this study, 3 miRNAs were differentially expressed, including miR-181b, miR-324 and miR-518b. In pre-clinical studies, miR-181b has been shown to promote cellular proliferation and reduce apoptosis in cervical cancer cells[32], mediate tumorigenesis through STAT3[33], and induce gemcitabine resistance in pancreatic cancer cells[34]. In human germ cell tumors, up-regulation of miR-518b was associated with a cisplatin-resistant phenotype[35]. These studies may explain the improved outcomes seen in our work that favored the *KRAS*-variant patients, as *KRAS-*variant tumors had lower expression levels of miR-518b. In a study of nasopharyngeal carcinoma, downregulation of miR-324 was associated with radioresistance[36]. In our study, *KRAS-*variant tumors had lower levels of miR-324, although there was no clear radioresistance as these patients had improved rates of local-regional control.

For a global view of what the differential miRNA expression found in our tumors may represent, we used miR System, which combines 7 algorithms and 2 validated databases to identify potential gene targets of miRNAs and their function, as well as pathway analysis using KEGG (Kyoto Encyclopedia of Genes and Genomes)[37]. The target genes of miR-181b include *MAP3K3* (mitogen-activated protein kinase 3, top hit), *ESR1* (estrogen receptor 1, a validated target), and *KRAS*, while those for miR-518b include *CTNNBIP1* (β-catenin interacting protein) and *MAP3K7IP3* (mitogen-activated protein kinase 7 interacting protein 3). For pathway analysis of the enriched target genes, differential expression of the miRNAs listed above were associated with long-term potentiation, pathways in cancer, and endometrial cancer, as well as the following signaling pathways: TGF-β, ErbB, MAPK, and Wnt. The Wnt/β-catenin signaling pathway has been shown to be dysregulated in 10–45% of endometrial cancers and is intimately regulated by estrogen and progesterone[38]. In addition, KRAS is an important upstream mediator of the MAPK pathway, and overexpression can lead to increased activation of the RAF/MEK/MAPK pathway and thus promote tumorigenesis. Previous work has shown that the MAPK pathway is activated in triple negative breast cancer with the *KRAS*-variant, and is associated with lower estrogen signaling[24]. These results and others indicate that the role of the *KRAS*-variant in cancer risk and biology may be dependent on hormonal environment such as menopausal status, an ongoing topic of study.

We also performed pathway analysis for the 10 differentially expressed miRNAs for tumors with and without lymphovascular invasion. Of interest, predicted pathways involved in focal adhesion, regulation of the actin cytoskeleton, gap junction and adherens junction were top hits by KEGG, as well as the Wnt, MAPK, TGF-β, and ErbB signaling pathways.

While our study was limited by the small sample size of the RTOG trials we utilized, which provided insufficient statistical power for us to develop solid conclusions regarding the potential role of the *KRAS-*variant in predicting endometrial cancer risk or outcome, we were able to see a potential association through altered miRNA expression in tumors. In addition, we were able to prospectively collect tissue samples in a cooperative group setting and perform a translational research study, which has produced preliminary data worth validating in larger cohorts. These findings further support the hypothesis that there are measurable ways to assess the heterogeneity of endometrial cancer which could provide important prognostic information in the future to enable practitioners to more appropriately and individually direct patient therapy.

## Supporting Information

Table S1 **Association between miRNA expression and lymphovascular invasion.**
(DOCX)

Table S2 **Association between miRNA expression and age.**
(DOCX)
