# Supplementary material for: The KRAS-Variant and miRNA Expression in RTOG Endometrial Cancer Clinical Trials 9708 and 9905
Source: PLoS One. 2014 Apr 14;9(4):e94167. doi: 10.1371/journal.pone.0094167 (PMC3986055; doi:10.1371/journal.pone.0094167)
Supplement: Table S1 — Association between miRNA expression and lymphovascular invasion. (DOCX) [file pone.0094167.s001.docx]

**Table S1. Association between miRNA expression and lymphovascular invasion**

| ID | logFC | p-Value | Adjusted p-Value | m.e. LVI Absent | m.e. LVI Present |
| --- | --- | --- | --- | --- | --- |
| hsa-miR-194 | 2.1114 | 0.0034 | 0.5265 | 7.2210 | 5.1097 |
| hsa-miR-192 | 1.5884 | 0.0101 | 0.6580 | 5.8903 | 4.3019 |
| hsa-miR-203 | 1.7951 | 0.0208 | 0.6580 | 4.7650 | 2.9699 |
| hsa-miR-345 | 1.1132 | 0.0223 | 0.6580 | 4.2558 | 3.1426 |
| hsa-miR-30e-3p | 0.8575 | 0.0290 | 0.6580 | 3.8185 | 2.9609 |
| hsa-miR-210 | 1.4213 | 0.0341 | 0.6580 | 4.1947 | 2.7734 |
| hsa-miR-20a | 0.7211 | 0.0385 | 0.6580 | 1.6235 | 0.9024 |
| hsa-miR-301 | 1.1265 | 0.0412 | 0.6580 | 7.6780 | 6.5514 |
| hsa-miR-34a | 1.0273 | 0.0502 | 0.6580 | 5.4249 | 4.3977 |
| hsa-miR-200a | 1.6225 | 0.0503 | 0.6580 | 7.2246 | 5.6021 |

Key: logFC = log fold change; m.e. = mean expression, LVI = lymphovascular invasion
