# Supplementary material for: The KRAS-Variant and miRNA Expression in RTOG Endometrial Cancer Clinical Trials 9708 and 9905
Source: PLoS One. 2014 Apr 14;9(4):e94167. doi: 10.1371/journal.pone.0094167 (PMC3986055; doi:10.1371/journal.pone.0094167)
Supplement: Table S2 — Association between miRNA expression and age. (DOCX) [file pone.0094167.s002.docx]

**Table S2. Association between miRNA expression and age**

| ID | logFC | p-Value | Adjusted p-Value | m.e. Age <51 years | m.e. Age >51 years |
| --- | --- | --- | --- | --- | --- |
| hsa-miR-20b | 2.0488 | 0.0089 | 0.9631 | 7.8980 | 5.8492 |
| hsa-miR-10a | 1.6580 | 0.0133 | 0.9631 | 3.5832 | 1.9252 |
| hsa-miR-187 | 2.3368 | 0.0185 | 0.9631 | 8.9820 | 6.6452 |
| hsa-miR-432 | -2.0513 | 0.0311 | 0.9798 | 6.6586 | 8.7099 |
| hsa-let-7d | -0.8405 | 0.0385 | 0.9798 | 3.7317 | 4.5722 |

Key: logFC = log fold change; m.e. = mean expression
